# A strategy for validation of variables derived from large-scale electronic health record data

**DOI:** 10.1016/j.jbi.2021.103879

**Published:** 2021-07-27

**Authors:** Lin Liu, Ranier Bustamante, Ashley Earles, Joshua Demb, Karen Messer, Samir Gupta

**Affiliations:** a VA San Diego Healthcare System, 3500 La Jolla Village Dr, San Diego, CA 92161, USA; b University of California San Diego, 9500 Gilman Dr, La Jolla, CA 92093, USA; c Veterans Medical Research Foundation, 3350 La Jolla Village Dr, San Diego, CA 92161, USA

**Keywords:** Electronic phenotyping, Large-scale electronic health records, Data abstraction validation, Sample size, Positive predictive value, Negative predictive value

## Abstract

**Purpose:**

Standardized approaches for rigorous validation of phenotyping from large-scale electronic health record (EHR) data have not been widely reported. We proposed a methodologically rigorous and efficient approach to guide such validation, including strategies for sampling cases and controls, determining sample sizes, estimating algorithm performance, and terminating the validation process, hereafter referred to as the San Diego Approach to Variable Validation (SDAVV).

**Methods:**

We propose sample size formulae which should be used prior to chart review, based on pre-specified critical lower bounds for positive predictive value (PPV) and negative predictive value (NPV). We also propose a stepwise strategy for iterative algorithm development/validation cycles, updating sample sizes for data abstraction until both PPV and NPV achieve target performance.

**Results:**

We applied the SDAVV to a Department of Veterans Affairs study in which we created two phenotyping algorithms, one for distinguishing normal colonoscopy cases from abnormal colonoscopy controls and one for identifying aspirin exposure. Estimated PPV and NPV both reached 0.970 with a 95% confidence lower bound of 0.915, estimated sensitivity was 0.963 and specificity was 0.975 for identifying normal colonoscopy cases. The phenotyping algorithm for identifying aspirin exposure reached a PPV of 0.990 (a 95% lower bound of 0.950), an NPV of 0.980 (a 95% lower bound of 0.930), and sensitivity and specificity were 0.960 and 1.000.

**Conclusions:**

A structured approach for prospectively developing and validating phenotyping algorithms from large-scale EHR data can be successfully implemented, and should be considered to improve the quality of “big data” research.

## 1. Introduction

Large-scale electronic health records (EHRs) contain a wide array of data that can be leveraged to conduct epidemiologic and quality improvement research [1,2]. Data abstraction algorithms are often used to extract variables from large-scale EHR data and can be used for a variety of purposes, such as to identify a study cohort [3–6] or define predictors and outcomes of interest [7–10]. This process is commonly referred as electronic phenotyping [11]. The accuracy and precision of results from analyses using the variable via electronic phenotyping depend heavily on the performance of the algorithms used to create the phenotype of interest.

Validation of electronic phenotyping is a major challenge. Validation typically consists of comparing the resulting phenotype against manual chart review as the reference standard [12,13]. However, this is logistically difficult when using large-scale EHR data because such datasets typically contain hundreds of thousands to millions of patients, with multiple variables of interest and multiple potential values for each variable. Reviewing more than a small fraction of records through manual chart review is not feasible, thus raising questions about the size and scope of the sample size required to achieve an accurate estimate of an electronic phenotyping algorithm’s performance.

A structured approach for validation of electronic phenotyping is required to ensure high quality research. The strategy should include an unbiased approach to 1) select a representative sample, 2) determine sample size required for review, 3) estimate phenotyping algorithm performance, and 4) define parameters for stopping iterative development once target performance is achieved. Sample size selection is a challenge in large-scale EHR datasets because the true value of the variable of interest is only knowable through chart review. In smaller scale datasets, true values are often known prior to validation, as is common in research focused on diagnostic tests [14,15]. In the setting of evaluating a diagnostic test with known true case and control status, sensitivity and specificity serve as the target performance measures (Table 1), and the minimum number of true cases and true controls required for review are determined by pre-specified targets for sensitivity and specificity [15,16].

Subsamples of true cases and true controls can be sampled separately from the study population. When the prevalence of cases is moderately common within the study population (e.g. 0.20–0.80), the total sample size of a random sample of the population can be estimated by accounting for the prevalence of the true cases, and the researcher can select a subsample with both true cases and true controls together [16,17]. However, when the prevalence of cases is extremely low or high in the study population (e.g. <0.10 or >0.90), using a random sample under the assumption that a sufficient number of true cases and controls will be included may not be an efficient or even feasible approach. Since achieving a sufficiently sized subsample of combined true cases and true controls could result in a need for a very large projected sample, manual chart review may incur substantial costs. An additional challenge for big data research pertains to estimation of sensitivity and specificity. When true case/control status is known prior to validation, sensitivity and specificity can be estimated directly from the validation sample. However, this approach is not feasible when using large-scale EHR data, because the true case or control status is often not known prior to review.

To address this challenge, some have chosen to review subsamples of *putative* cases and *putative* controls ranging from 50 to 1,200 as identified by an electronic phenotyping algorithm instead [8,18–21]. For example, Mamtani and colleagues identified 18,000 putative bladder cancer cases from a database with over 11 million patients, and then manually reviewed a random sample of 210 putative bladder cancer cases to assess the performance of the phenotyping algorithm using positive predictive value (PPV) [8]. Nadkarni et al. (2014) validated an algorithm that abstracted chronic kidney disease status using a random sample of 600 predicted disease and non-disease patients, respectively, for the primary study site, and then reduced the sample size to 25 disease and 25 non-disease patients for the secondary site [21]. Both PPV and negative predictive value (NPV) were used to examine the algorithm performance. To examine the performance of an EHR based phenotyping algorithm of community associated methicillin-resistant *Staphylococcus aureus*, Jackson et al. (2016) selected a random sample of 25 to 50 potential cases and controls for each site for chart review and estimated the PPV and NPV [20]. Although the strategy of selecting putative cases and controls has been used for these and other studies in practice, from our literature review, there are no published methods to provide standardized guidance on how to select subsamples and determine the sample size for validation of a phenotyping algorithm when using large-scale EHR data with an unknown case/control prevalence [20–23]. In this scenario, PPV and NPV would be selected as the primary performance measures.

As previously mentioned, the sampling strategy, sample size and performance criteria should be specified prior to chart review using well-established statistical principles. Without such pre-specification, sample sizes may be too small, resulting in imprecise performance estimates with a large variance (a wide confidence interval), or alternatively sample sizes may be unnecessarily large, resulting in inefficient and labor intensive chart reviews. Furthermore, an electronic phenotyping algorithm usually needs to go through several development and validation iterations [24] before achieving the target performance. These iterations might include modification of the phenotyping algorithm, lowering the target performance values and/or increasing the sample size to improve precision of the performance estimates. To our knowledge, there are no published approaches to guide how this stepwise process might be conducted, or how many iterations of the development and validation cycle are required. A pre-specified approach is desirable in order to avoid potential early cessation of algorithm development when informal “favorable” performance estimates are produced. Additionally, a pre-specified approach can also avoid continued, labor intensive chart review when additional sampling alone is unlikely to achieve adequate performance, and a new development cycle is needed. Finally, we postulate a need to establish pre-specified well thought out criteria for stopping for both success and for failure after an independent validation step, in order to produce more reliable performance estimates at the end of the development and validation process. Development of novel methodology is necessary, given the burgeoning use of large-scale EHR data for research and the need to ensure that results are optimally interpreted.

In this paper, we propose a methodologically rigorous and efficient approach for the validation of phenotypes derived from large-scale EHR data using PPV and NPV as performance measures, hereafter referred to as the San Diego Approach for Variable Validation (SDAVV). Specifically, we outline 1) a sampling strategy for cases and controls, 2) minimum sample size required to achieve target performance estimates, 3) an approach to estimate phenotyping algorithm performance, and 4) a stepwise process for validating variables of interest. As illustrative examples, we applied the SDAVV to validate two phenotyping algorithms, one for identifying normal colonoscopy cases and abnormal colonoscopy controls, and one for identifying aspirin exposure within the Department of Veterans Affairs (VA) healthcare system utilizing a large national dataset encompassing over two million records and 15 years of data.

## 2. Methods

### 2.1. Sampling strategy and preliminary estimates

We first determined the sampling strategy, selected the primary performance measures to be used to validate the phenotyping algorithm, and pre-specified the target performance of the measures. The primary performance measures were PPV and NPV. PPV was defined as the proportion of true cases (as identified by blinded manual chart review) among all those classified as putative cases by the phenotyping algorithm, and NPV as the proportion of true controls among those classified as putative controls by the phenotyping algorithm. Since the true cases and true controls were unknown before the chart review, we planned to use random sampling of putative cases and putative controls, as identified by the electronic phenotyping algorithm. Once the phenotyping algorithm was judged to have sufficient performance (or not amenable to improved performance) within the current development cycle [24], we estimated prevalence of cases as identified by the algorithm. Then, we determined the minimum sample size required for manual chart review using an estimated or assumed true PPV/NPV, and a pre-specified target critical value, as is described in further detail below.

### 2.2. Determine sample size

Target performance was set by requiring the one-sided *α*-level lower confidence bounds for the estimated population PPV and NPV to be above a pre-specified critical threshold *p*_0_, as the criterion for a successful validation. The number of putative cases and putative controls needed for review was then determined. Since two potentially correlated performance measures were estimated, a Bonferroni correction was used for multiple comparison adjustment to ensure an overall confidence of (1 −*α*)%. Therefore, the one-sided (1 −*α/*2)% confidence lower bounds for the population PPV/NPV would be calculated as

(1) $$\hat{p}-z_{\alpha /2}\sqrt{\hat{p}(1-\hat{p})/n}$$

where $\hat{p}$ is the estimated PPV/NPV from the to-be reviewed sample, *n* is the number of putative cases and putative controls required for review, and *z*_*α/*2_ is the critical value of the standard normal distribution. If *α* = 0.05, then the critical value *z*_.025_ = 1.96 would be used because of the Bonferroni correction. Now, we select our best guess for an assumed or anticipated target PPV/NPV of our phenotyping algorithm, perhaps using a current estimate from a current validation sample and substitute this for $\hat{p}$ in formula (1). In order for the confidence lower bounds in formula (1) to lie above a critical value of *p*_0_, the number of putative cases and putative controls needed for chart review would be

(2) $$n=\frac{z_{\propto /2}^{2}\hat{p}(1-\hat{p})}{{\left(\hat{p}-p_{0}\right)}^{2}}$$

The difference between sample PPV/NPV and the critical threshold of the lower bounds for estimated population PPV/NPV $\left(\hat{p}-p_{0}\right)$ is called the margin of error. To demonstrate how to use formula (2), let us assume the anticipated PPV/NPV are 0.950. If we set the critical lower bounds at 0.900 (equivalent to the margin of error at 0.05), we would estimate the needed sample size as *n* = 1.96^2^*0.950*(1 − 0.950) ÷ (0.950 − 0.900)^2^ = 73 using formula (2). Thus, a minimum of 73 putative cases and 73 putative controls would be required for review.

Table 2 presents a range of reasonable sample sizes for chart review (100−250) and critical lower bounds for a range of anticipated PPV/NPVs (0.850−0.950). For example, if 100 putative cases and 100 putative controls were randomly sampled and the anticipated PPV/NPV were both 0.950 or above, then the critical lower bounds for the population PPV/NPV would be 0.907 or above. If the validation was successful, the lower bound of the estimated PPV/NPV would lie above 0.907 and we would claim with 95% confidence that population PPV/NPV are both greater than 0.907. Furthermore, if the true PPV/NPV are at least as great as anticipated, then at the computed sample size the validation study has a 95% chance of being successful. Table 2 can be easily expanded using formula (1) above to provide projections of critical lower bounds for a wider range of sample sizes and/or anticipated PPV/NPVs.

### 2.3. Estimate phenotyping algorithm performance

Phenotyping algorithm performance was summarized primarily using the selected performance measures PPV and NPV, and their one-sided (1 −*α/*2)% confidence lower bounds. Successful validation was declared if the confidence intervals for both measures were above the pre-specified critical lower bounds. After completing manual chart review, PPV and NPV were estimated directly from the subsample of putative cases and putative controls. Sensitivity and specificity could also be estimated, given that these have been recommended as performance measures when validating discrete variables of interest [25]. Sensitivity was defined as the proportion classified as putative cases by the algorithm among the true cases, and specificity as the proportion classified as putative controls by the phenotyping algorithm among the true controls. Sensitivity and specificity could not be directly estimated from the subsample of putative cases and controls. However, they could be calculated by combining PPV and NPV with the prevalence of cases identified by the phenotyping algorithm (*w*) as follows:

(3) $$\text{Sensitivity}=\frac{PPV*w}{PPV*w+\left(1-NPV\right)*(1-w)}$$

(4) $$\text{Specificity}=\frac{NPV*(1-w)}{NPV*(1-w)+\left(1-PPV\right)*w}$$

Computations of sensitivity and specificity using Bayes’ theorem [26] are provided in Appendix A. In practice, PPV and NPV would be estimated from the reviewed samples, and the prevalence of cases identified by the phenotyping algorithm over the study population (*w*) could be calculated directly.

We explored possible values for sensitivity and specificity given a wide range of PPV/NPV (0.850–0.990) and prevalence of cases identified by a phenotyping algorithm (0.05–0.95). Only values for sensitivity are shown in Table 3 below since specificity is inversely related to sensitivity with the prevalence of 1 – *w* (see Appendix B for specificity values). We found that for a moderate prevalence between 0.20 and 0.80, and a PPV and NPV of 0.90 or above, that estimated sensitivity and specificity would both be ≥ 0.692. If higher sensitivity and specificity are required, a higher PPV/NPV should be targeted when developing the phenotyping algorithm.

### 2.4. Stepwise validation process

Using Table 2 and Table 3 as guides, we propose a stepwise validation process in which we estimate sample PPV and NPV during the phenotyping algorithm development stage, set the critical lower bounds for PPV/NPV, and then identify the validation sample size that could achieve the closest value to the target lower bounds in Table 2. For simplicity, Table 2 includes feasible sample sizes commonly used in clinical research, but the exact sample size for each step could be calculated directly using formula (2). If the lower bounds of PPV/NPV estimated from the reviewed sample do not reach their target values, we cannot claim that population PPV/NPV are greater than the lower bound thresholds with 95% confidence, and we would conclude inadequate phenotyping algorithm performance at this stage. If considerations suggest the phenotyping algorithm could be improved, we would propose modification of the algorithm with another round of review.

During the second round of review, we would also consider lowering the targets, increasing the sample size, or implementing both simultaneously and then continuing until the desired targets were reached or further improvement was not feasible. If the initial targets were missed because the phenotyping algorithm was unlikely to achieve them even after modification, lowering the targets is recommended. If the initial targets were missed because the lower bound was not estimated efficiently due to small sample size of the reviewed sample, increasing the number of cases and controls for the next round of review is recommended. To be conservative, we could also adjust both. The following is a step-by-step example of the proposed process.

1. **Set initial critical lower bounds and determine minimum sample size required.** If anticipated PPV and NPV during the algorithm development stage were 0.950, we would set the initial critical lower bounds at 0.900. According to Table 2, we would manually review 100 putative cases and 100 putative controls. If the estimated PPV and PPV from the reviewed samples reach 0.95 or greater, the lower bounds of PPV/NPV would be at least 0.907 and the initial critical lower bounds of 0.900 would be achieved, indicating successful validation at the first step.
2. **If initial critical lower bounds are not met, modify phenotyping algorithm, lower critical lower bounds, and increase sample size.** If critical lower bounds were not reached during the first iteration, we would simultaneously lower the critical lower bounds and increase the sample size during the second round of review. If anticipated PPV/NPV were lowered to 0.900, we would set the revised target lower bounds at 0.850 (if clinically acceptable). According to Table 2, we would manually review 150 putative cases and 150 putative controls. If the estimated PPV/NPV from the reviewed samples reach 0.900 or greater, the resulting lower bounds of PPV/NPV would be at least 0.852 and the revised critical lower bounds of 0.850 would be achieved, again indicating successful validation at the second step.
3. **Repeat process until critical lower bounds are reached or further improvement is not feasible.** If the critical lower bounds were not reached, we would continue to modify the algorithm, lower the critical lower bounds, increase the sample size, and complete another iteration until the desired targets were reached or further improvement was not feasible.
4. **Calculate all four performance measures and the validation process is completed.** Finally we would calculate PPV, NPV, sensitivity and specificity with lower one-sided 95% confidence intervals and the validation process would be completed.

Note: Because of the sequential nature of the validation process, multiple testing would inflate the family-wise error rate such that the nominal confidence level for the 95% confidence interval would not hold as expected if two or more steps were conducted.

## 3. Illustrative examples

We applied the SDAVV described above to validate two phenotyping algorithms – one for identifying normal colonoscopy cases and abnormal colonoscopy controls, and one for identifying aspirin exposure using a combination of structured medication data and unstructured free-text progress notes – within the VA healthcare system.

### 3.1. Identifying normal colonoscopy cases and abnormal colonoscopy controls

In this first illustrative example, we implemented the SDAVV to validate an approach for identifying normal colonoscopy cases and abnormal colonoscopy controls.

#### 3.1.1. Sampling strategy and preliminary estimates

**Fig. 1.**
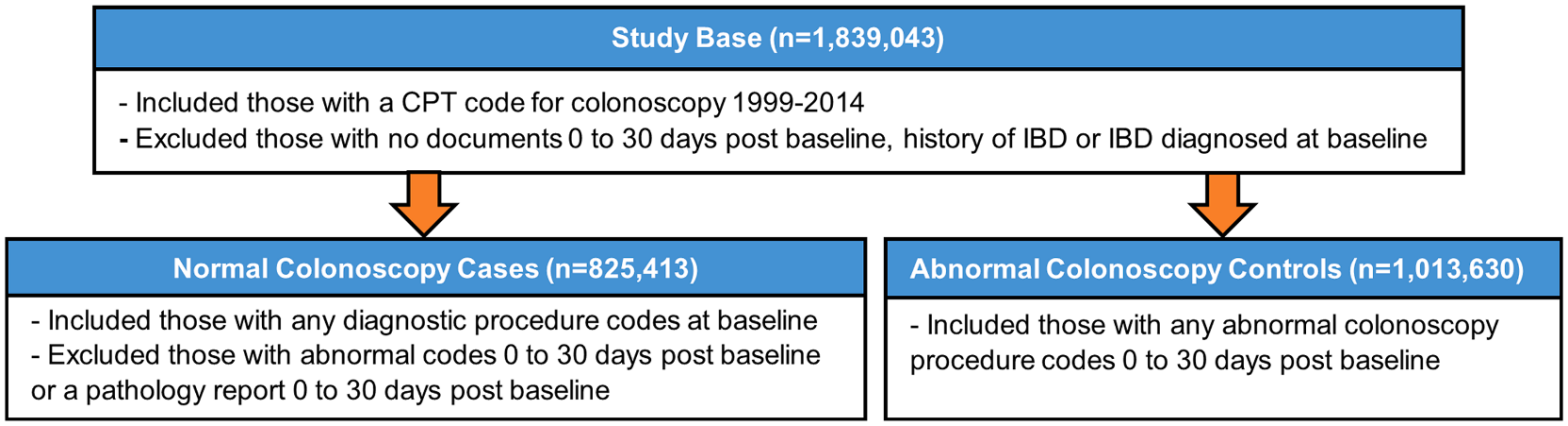
Selection Criteria for Illustrative Example of Normal Colonoscopy Cases and Abnormal Colonoscopy Controls. **Abbreviations:** CPT, Current Procedural Terminology: IBD, Inflammatory Bowel Disease.

Our study base, which has been previously described [27], consisted of 1,839,043 Veterans with at least one Current Procedural Terminology (CPT) code for colonoscopy from 1999 to 2014, after excluding patients with no documentation of colonoscopy on the day of their CPT code up to 30 days after that initial code and patients with history or a diagnosis of inflammatory bowel disease at the date of initial code. We identified the baseline procedure date and applied relevant exclusion criteria (see Fig. 1 for a full outline of the selection criteria; details for exclusion criteria are included in Appendix C). Normal colonoscopy was defined as no polyps removed or biopsies taken; abnormal colonoscopy was defined as any polyps removed or biopsies taken. The algorithm resulted in 825,413 putative cases and 1,013,630 putative controls. Prevalence of normal colonoscopy as identified by the algorithm was 0.449.

#### 3.1.2. Determine sample size

During the phenotyping algorithm development phase, we used an initial rule-based approach and worked toward finding an appropriate definition to build up to a finalized version of the algorithm by randomly reviewing a small number of charts and modifying the approach. Based on this process, we anticipated that we could achieve a PPV and NPV of 0.950. Following the validation process proposed above, we set the initial critical lower bounds at 0.900 and randomly sampled 100 putative cases and 100 putative controls.

#### 3.1.3. Estimate phenotyping algorithm performance

Reviewers (RB and AE) manually reviewed 100 putative cases and 100 putative controls in random order, and a clinician with expertise in the entity of interest (SG) spot checked charts for accuracy. Performance measures were estimated after the review was completed (Table 4). Sample PPV and NPV were both 0.970 and the 95% confidence lower bounds of population PPV/NPV were 0.915. Sample PPV/NPV were then combined with the prevalence of normal colonoscopy cases as identified by the algorithm to calculate sensitivity and specificity using formulas (3) and (4), which were 0.963 and 0.975, respectively.

#### 3.1.4. Stepwise validation process

**Fig. 2.**
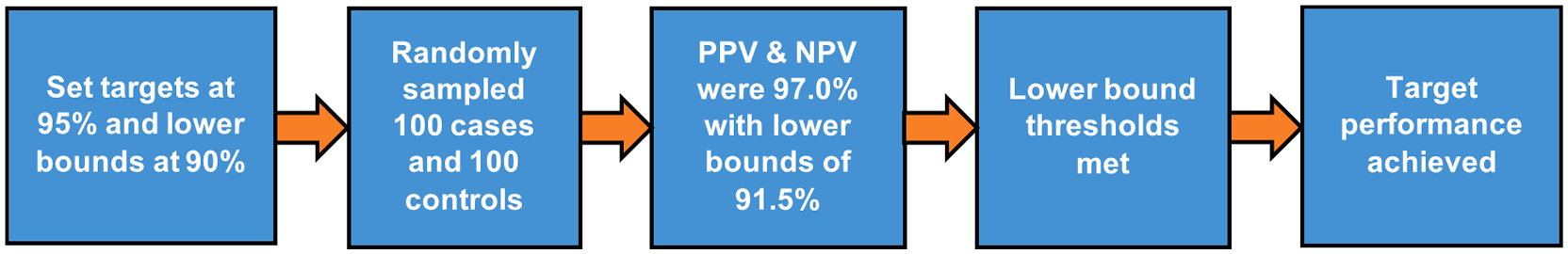
Illustrative Example of Normal Colonoscopy Cases and Abnormal Colonoscopy Controls. **Abbreviations:** NPV, negative predictive value; PPV, positive predictive value.

According to the approach laid out above, we could claim that population PPV/NPV of the algorithm were both greater than 0.915 (better than the critical lower bound of 0.900) with 95% confidence. Thus, we concluded that the algorithm performed well with both high PPV for identifying cases with normal colonoscopy and high NPV for identifying individuals with abnormal colonoscopy and stopped the validation process. A summary of how we applied the SDAVV to the first illustrative example was summarized in Fig. 2.

### 3.2. Identifying aspirin exposure

In another illustrative example, we implemented the proposed method to validate an approach for ascertaining aspirin exposure using both structured medication data and unstructured free-text progress notes in a cohort of individuals exposed to colonoscopy [28]. The critical threshold for PPV and NPV was selected at 0.90 based on the estimates from the algorithm development phase. We developed a phenotyping strategy using unstructured free-text data only and found that the lower bound for PPV, which was 0.89, did not achieve the critical threshold 0.90 during the first round of chart review validation (Table 5). In the second iteration, we modified the algorithm by adding the structured data, and maintained the goal of achieving target performance for PPV at 0.90. We found that the estimated lower bounds of PPV and NPV were both above 0.90 at this round of chart review, so we concluded that the algorithm performed well and stopped the validation process. Combined with the estimated prevalence of aspirin exposure 0.36, the sensitivity and specificity were estimated to be 0.96 and 1.00, respectively. The details of aspirin exposure phenotyping algorithm were reported in Bustamante et al. (2019) [28].

## 4. Discussion

Validation of electronic phenotyping algorithms developed for large-scale EHR data is a challenge. In large-scale EHR studies where the true value of the phenotype variable is unknown, we propose a methodologically rigorous and efficient iterative approach, known as the SDAVV, for validating electronic phenotyping algorithms by sampling putative cases and putative controls for review. Implementing a pre-specified sample size selection approach, based on the performance measures for PPV/NPV estimated during phenotyping algorithm development, before initiating manual chart review, has the advantage of improving efficiency and reducing risk for potential bias by avoiding inefficient review of an unnecessarily large number of charts, and avoiding a smaller than needed sample that results in a wide confidence interval around performance estimates. Our approach addresses a gap in the literature, given that in other validation of electronic phenotyping algorithms, there was a lack of standardized methods in describing the number of cases and controls to review [20,21,23]. Indeed, sample sizes utilized for chart review to validate electronic phenotyping algorithms reported in the literature range from 50 to 1,200 without an accompanying formal sample size projection. Our proposed sampling strategy ensures that a sufficient yet parsimonious sample of putative cases and putative controls are selected for review even if the prevalence of cases within the study population is extremely low or high (e.g. < 0.10 or greater than 0.90). Algorithm development and validation works best as an iterative process [23]. Within this process, these iterations might include modification of the phenotyping algorithm as well as lowering the target performance values. Our approach includes statistical rules for stopping the iterative process of algorithm development and validation. To our knowledge, this is the first structured approach to incorporate a stepwise validation process within phenotyping algorithm development. We postulate that implementation of our proposed approach has the potential to reduce bias and improve research efficiency, filling a gap in phenotyping algorithm validation methodology, and improving the quality of electronic phenotyping.

An advantage of our proposed approach is that it is highly adaptable (Table 6). First, both PPV and NPV were selected as the primary performance measures to ensure that the phenotyping algorithm was validated among both putative cases and putative controls. Bonferroni correction was applied for multiple comparisons adjustment of two primary measures, which affects their confidence intervals (PPV and NPV). However, others may choose to modify the approach and only sample putative cases to estimate PPV if only identifying true cases is of interest as in Mamtani’s study [8], such that Bonferroni correction would not be needed.

Second, the initial PPV/NPV and lower bounds could also be adjusted, and the sample size could be still calculated using formulas (1) and (2), and sensitivity and specificity would be calculated using formulas (3) and (4). Third, based on the results from the algorithm development stage, others may choose to use different estimates and target different lower bounds for PPV and NPV, respectively, which would result in a different sample sizes for putative cases and putative controls. To simplify our approach, we chose to randomly sample an equal number of putative cases and putative controls. PPV and NPV are complementary, so refining the phenotyping algorithm would change both performance measures. To reduce bias, we recommend resampling both putative cases and putative controls if either PPV or NPV fail to hit their targets. Since the original sample is based on the previous algorithm and the total number of putative cases and putative controls is likely to change after the modification, it is inappropriate to assess new performance measurements using the original sample because this could cause overfitting and therefore bias toward favorable performance. Another reason to resample both putative cases and putative controls is to keep reviewers blinded to case/control status. If we reuse the sample putative cases and controls, reviewers may recall the true case/control status, and the resulting adjudication of case/control status would not be independent of the prior review, and potentially be subject to bias.

The approach is also adaptable for scenarios which require additional iterations in the validation process if the initial critical lower bounds were not met. However, this decision often depends on time and resources available. As we pointed out earlier, since validation is a stepwise process, the nominal confidence level for the 95% confidence intervals might not hold, without a pre-specified number of iterative steps, the correction for multiple testing becomes challenging. If the maximum number of iterative steps could be pre-specified, Bonferroni correction could be applied and the sample size in each step will be larger than what we have proposed in the paper. In practice, we suggest reviewing a new independent sample after the last round of the validation, and reporting the performance measures using this new sample such that the 95% confidence level can be maintained. Another iterative process which may be used as a way to adapt our validation process is sequential testing. Rather than using independent samples where we may modify our algorithm between samples, sequential testing would allow for collecting data sequentially until the goal is reached [29–32]. There is uncertainty in how to apply our modified algorithm during this process, and additional work needs to be completed to determine the feasibility and efficacy of this method.

The proposed approach can be applied to a variety of different scenarios and is highly generalizable (Table 7). For example, the approach can be used to identify a study cohort, define a predictor, or validate an outcome of interest [3–10]. The approach is particularly useful for validating rare outcomes such as colorectal cancer [10]. When validating phenotypes with low prevalence, strategic sampling of cases and controls is often the only feasible design [14,33]. Our approach will ensure that enough cases/controls are reviewed. Although our illustrative examples focused on binary variables, our approach could be easily modified to validate continuous and categorical phenotypes as well. In order to validate continuous phenotypes (e.g. weight) using the proposed process, an acceptable range for error could be set beforehand (e. g. within 10 lb of the true value). In order to validate categorical phenotypes (e.g. smoking), we recommend validating each category individually and comparing one category (e.g. current smoker) vs. all others. Finally, our approach could be used to validate phenotyping algorithms using structured, unstructured free-text data, or a combination of both data types [34] as shown in the illustrative examples. The algorithm could be rule-based, machine learning-based [24], model-based [35] as well as natural language processing (NLP) algorithms derived from free-text data at the mention-level, document-level, or event-level [36]. In NLP specifically, we are oftentimes only interested in positive outcomes, and these are cases in which negative/missing are considered negative. In this scenario, we would validate positive outcomes against negative and missing combined.

Often, the outcomes of sensitivity and specificity are of interest for a novel electronic phenotyping algorithm developed for a specific variable, such as presence/absence of a specified disease or characteristic. Sensitivity and specificity can be easily derived and calculated from the sample PPV/NPV and the prevalence of cases identified by the algorithm. There are situations where phenotyping algorithm development must be driven by sensitivity and/or specificity, although it is noted as a significant challenge in validating the phenotype algorithm without knowing the true status in the large scale EHR setting. In these scenarios, we recommend using sensitivity and specificity as a reference to select the target PPV and NPV and applying the proposed methodology. However, since sample PPV/NPV will be combined with estimated prevalence of cases identified by the algorithm to calculate sensitivity and specificity, these estimates will vary widely based on the estimated PPV/NPV and the prevalence of the algorithm. As seen in Table 3, for events with an extremely low prevalence within the study population, a high target PPV and NPV should be considered in order to reach adequate estimated sensitivity and specificity. For example, if study population prevalence is 0.20 with a goal of achieving 90% sensitivity, a higher target for PPV, such as 0.97 instead of 0.95, should be selected.

Our proposed method may have some limitations to consider. First, selecting an initial target is a subjective assessment in many cases and there is no formal approach to determine a threshold as a good and acceptable PPV/NPV. We have recommended selecting the initial PPV and NPV either based on previous research or the potential estimates during the algorithm development stage of the phenotype. Additionally, with some specific phenotypes, there are repositories of electronic phenotypes that report performance such as Phenotype KnowledgeBase (PheKB) [37]. PheKB publishes performance data, such as PPV, that can be used during our algorithm development step for establishing an achievable lower bound performance statistic. For example, the validated PPVs for PheKB’s algorithm to define the colorectal cancer (CRC) cases were 0.86, 0.92, and 1.00 from three implementation sites [38]. If a new phenotyping algorithm is desired for defining CRC cases, one can consider setting the lower bound of PPV in the range of 0.86–1.00. Second, a common challenge in the development of electronic phenotyping algorithms is how to handle unclassified outcomes that are not clearly positive or negative cases. In this scenario, the validation approach is not the same as the validation of a simple diagnostic test result that always yields a definite positive or negative result [23], where sensitivity, specificity, PPV and NPV can all be easily generated. Having a test or electronic phenotyping algorithm that results in a third category of unclassified subjects, in addition to positive and negative results, changes the way we interpret and calculate the performance statistics. Our current approach can provide all these estimates if we restrict the validation to subjects classified either as definite (positive) cases or definite (negative) controls. To validate the overall performance on classifying patients as one of three categories (cases, controls, or unclassified), the validation could be conducted for three categories separately as previously discussed, with PPV being estimated for each category.

## 5. Conclusion

In conclusion, we developed a methodologically sound process to guide the rigorous validation of electronic phenotyping algorithms. We laid out a sampling strategy, sample size determination, estimation of algorithm performance, and a stepwise validation process. Then we applied the SDAVV to two phenotyping algorithms – one for identifying normal colonoscopy cases and abnormal colonoscopy controls, and one for identifying aspirin exposure using both structured medication data and unstructured free-text progress notes – within the VA healthcare system. The phenotyping algorithm for normal colonoscopy cases and abnormal colonoscopy controls resulted in 825,413 putative cases and 1,013,630 putative controls. PPV and NPV were both 0.970 and sensitivity and specificity were 0.963 and 0.975, respectively. The phenotyping algorithm for identifying aspirin exposure reached a PPV of 0.950, an NPV of 0.980, and sensitivity and specificity were 0.960 and 1.000. Based on these results, we postulate that implementing our proposed strategies for validating electronic phenotyping algorithms may reduce bias within and improve efficiency of research using large scale EHR data.

## Supplementary Material

Supplementary Material

## Figures and Tables

**Table 1:** Performance Measures Used to Evaluate Accuracy of Data Abstraction Algorithms.

		Chart Review		Performance Measures
		True Cases	True Controls	
	**Putative Cases**	True Positives (TP)	False Positives (FP)	$\text{PPV}=\frac{\text{TP}}{\text{TP}+\text{FP}}$
**Algorithm**	**Putative Controls**	False Negatives (FN)	True Negatives (TN)	$\text{NPV}=\frac{\text{TN}}{\text{FN}+\text{TN}}$
**Performance Measures**		$\text{Sensitivity}=\frac{\text{TP}}{\text{TP}+\text{FN}}$	$\text{Specificity}=\frac{\text{TN}}{\text{FP}+\text{TN}}$	

**Abbreviations:** NPV, negative predictive value; PPV, positive predictive value.

**Table 2:** Critical Lower Bounds Given a Range of Sample Sizes and Anticipated PPV/NPV.

Sample Size	Anticipated PPV/NPV	Critical Lower Bound	Sample Size	Anticipated PPV/NPV	Critical Lower Bound	Sample Size	Anticipated PPV/NPV	Critical Lower Bound
100	0.950	0.907	150	0.950	0.915	250	0.950	0.923
100	0.925	0.873	150	0.925	0.883	250	0.925	0.892
100	0.900	0.841	150	0.900	0.852	250	0.900	0.863
100	0.875	0.810	150	0.875	0.822	250	0.875	0.834
100	0.850	0.780	150	0.850	0.793	250	0.850	0.806

**Abbreviations:** NPV, negative predictive value; PPV, positive predictive value.

**Table 3:** Sensitivity Values Given a Wide Range of PPV/NPV and Prevalence.*

	PPV/NPV						
Prevalence (*w*)	0.850	0.880	0.900	0.920	0.950	0.970	0.990
0.95	0.991	0.993	0.994	0.995	0.997	0.998	0.999
0.90	0.981	0.985	0.988	0.990	0.994	0.997	0.999
0.80	0.958	0.967	0.973	0.979	0.987	0.992	0.997
0.70	0.930	0.945	0.955	0.964	0.978	0.987	0.996
0.60	0.895	0.917	0.931	0.945	0.966	0.980	0.993
0.50	0.850	0.880	0.900	0.920	0.950	0.970	0.990
0.40	0.791	0.830	0.857	0.885	0.927	0.956	0.985
0.30	0.708	0.759	0.794	0.831	0.891	0.933	0.977
0.20	0.586	0.647	0.692	0.742	0.826	0.890	0.961
0.10	0.386	0.449	0.500	0.561	0.679	0.782	0.917
0.05	0.230	0.278	0.321	0.377	0.500	0.630	0.839

**Abbreviations:** NPV, negative predictive value; PPV, positive predictive value.

* For simplicity, we assumed equal PPV and NPV in our calculation.

**Table 4:** Performance Measures for Illustrative Example of Normal Colonoscopy Cases and Abnormal Colonoscopy Controls.

	Prevalence	Chart Review		Performance Measures
Algorithm	n (%)	Normal	Abnormal	Estimate (LB*)
Normal	825,413 (44.9)	97	3	PPV = 0.970 (0.915)
Abnormal	1,013,630 (55.1)	3	97	NPV = 0.970 (0.915)

**Abbreviations:** LB, lower bound; NPV, negative predictive value; PPV, positive predictive value.

* The one-sided exact binomial confidence lower bound.

**Table 5:** Performance Measures for Illustrative Example of Aspirin Exposure.

Iteration Step	Strategy	PPV (LB*)	NPV (LB*)
1	Unstructured data	0.95 (0.89)	0.98 (0.93)
2	Unstructured and structured data	0.99 (0.95)	0.98 (0.93)

**Abbreviations:** NPV, negative predictive value; PPV, positive predictive value; LB, lower bound.

* The one-sided exact binomial confidence lower bound.

**Table 6:** Potential modifications to the SDAVV.

Select PPV as primary performance measure and randomly sample putative cases only Adjust estimated performance measures, target lower bounds, and/or sample size required Set different targets for PPV and NPV and select different sample sizes for cases and controls Adjust the number of iterations

**Abbreviations:** NPV, negative predictive value; PPV, positive predictive value; SDAVV, San Diego Approach to Variable Validation.

**Table 7:** Potential applications for the SDAVV.

Purpose	Types of Variables	Types of EHR Data
- Identify a study cohort (e.g. patients with colonoscopy)	- Binary (e.g. aspirin exposure)	- Structured (e.g. claims-based data)
- Define a predictor (e.g. aspirin exposure)	- Continuous (e.g. weight)	- Free-text (e.g. natural language processing)
- Define an outcome (e.g. colorectal cancer)	- Categorical (e.g. smoking)	- Combination (e.g. aspirin exposure)

**Abbreviations:** EHR, electronic health record; SDAVV, San Diego Approach to Variable Validation.

## Abbreviations:

EHRs: electronic health records
NPV: negative predictive value
PPV: positive predictive value
VA: Department of Veterans Affairs
SDAVV: San Diego Approach to Variable Validation
