# Supplementary material for: A strategy for validation of variables derived from large-scale electronic health record data
Source: J Biomed Inform. Author manuscript; Available in PMC 2022 Oct 28. (PMC9615095; doi:10.1016/j.jbi.2021.103879)
Supplement: Supplementary Material [file NIHMS1841350-supplement-Supplementary_Material.docx]

**Appendix A. Sensitivity and Specificity Derivation**

Sensitivity is the conditional probability $P(Algorithm+|Review+)$ or the probability that a subject is identified as a putative case by the algorithm given that they are a true case.

Using Bayes’ theorem, sensitivity can be calculated as follows:

| Sensitivity | $=P\left( Algorithm+ \vert Review+ \right)$ |
| --- | --- |
|  | $=\frac{P(Algorithm+ \cap Review+)}{P(Review+)}$ |
|  | $=\frac{P\left( Review+ \vert Algorithm+ \right)P\left( Algorithm+ \right)}{P\left( Review+ \cap Algorithm+ \right)+(P(Review+ \cap Algorithm -)}$ |
|  | $=\frac{P\left( Review+ \vert Algorithm+ \right)P(Algorithm+)}{P\left( Review+ \vert Algorithm+ \right) P\left( Algorithm+ \right)+P\left( Review+ \vert Algorithm- \right)P(Algorithm-)}$ |
|  | $=\frac{PPV*P(Algorithm+)}{PPV*P\left( Algorithm+ \right)+\left( 1-NPV \right)*P(Algorithm-)}$ |
|  | $=\frac{PPV*w}{PPV*w+\left( 1-NPV \right)*(1-w)}$ |

Specificity is the conditional probability $P\left( Algorithm- | Review- \right)$ or the probability that a subject is identified as a putative control by the algorithm given that they are a true control.

Specificity can be calculated as follows:

| Specificity | $=P\left( Algorithm- \vert Review- \right)$ |
| --- | --- |
|  | $=\frac{P(Algorithm- \cap Review-)}{P(Review-)}$ |
|  | $=\frac{P\left( Review- \vert Algorithm- \right) P(Algorithm-)}{P\left( Review- \cap Algorithm+ \right)+(P(Review- \cap Algorithm -)}$ |
|  | $=\frac{P\left( Review- \vert Algorithm- \right)P(Algorithm -)}{P\left( Review- \vert Algorithm- \right)P\left( Algorithm- \right)+P\left( Review- \vert Algorithm+ \right)P(Algorithm +)}$ |
|  | $=\frac{NPV*P(Algorithm-)}{NPV*P\left( Algorithm- \right)+\left( 1-PPV \right)*P(Algorithm+)}$ |
|  | $=\frac{NPV*(1-w)}{NPV*\left( 1-w \right)+\left( 1-PPV \right)*w}$ |

**Appendix B. Specificity Values Given a Wide Range of Sample PPV/NPV and Prevalence**

|  | **Sample PPV/NPV* (**$\hat{\boldsymbol{p}}$**)** | | | | | | |
| --- | --- | --- | --- | --- | --- | --- | --- |
| **Prevalence** $\boldsymbol{(w)}$ | 0.850 | 0.880 | 0.900 | 0.920 | 0.950 | 0.970 | 0.990 |
| 0.90 | 0.981 | 0.985 | 0.988 | 0.990 | 0.994 | 0.782 | 0.917 |
| 0.80 | 0.958 | 0.967 | 0.973 | 0.979 | 0.987 | 0.890 | 0.961 |
| 0.70 | 0.930 | 0.945 | 0.955 | 0.964 | 0.978 | 0.933 | 0.977 |
| 0.60 | 0.895 | 0.917 | 0.931 | 0.945 | 0.966 | 0.956 | 0.985 |
| 0.50 | 0.850 | 0.880 | 0.900 | 0.920 | 0.950 | 0.970 | 0.990 |
| 0.40 | 0.791 | 0.830 | 0.857 | 0.885 | 0.927 | 0.980 | 0.993 |
| 0.30 | 0.708 | 0.759 | 0.794 | 0.831 | 0.891 | 0.987 | 0.996 |
| 0.20 | 0.586 | 0.647 | 0.692 | 0.742 | 0.826 | 0.992 | 0.997 |
| 0.10 | 0.386 | 0.449 | 0.500 | 0.561 | 0.679 | 0.997 | 0.999 |
| 0.05 | 0.991 | 0.993 | 0.994 | 0.995 | 0.997 | 0.998 | 0.999 |
| 0.01 | 0.998 | 0.999 | 0.999 | 0.999 | 0.999 | 1.000 | 1.000 |
| **Abbreviations:** NPV, negative predictive value; PPV, positive predictive value.  *For simplicity, we assumed equal PPV and NPV in our calculation. | | | | | | | |

**Appendix C. Inclusion and Exclusion Criteria for Illustrative Example of Normal Colonoscopy Cases and Abnormal Colonoscopy Controls**

| **Group** | **Explanation** | **Identifiers** | **Codes** |
| --- | --- | --- | --- |
| Study base  (n=1,839,043) | Included those with a CPT code for colonoscopy 1999-2014  Excluded patients with no documentation of colonoscopy on the day of their CPT code up to 30 days after that initial CPT colonoscopy code | CPT codes for colonoscopy | 44388-44394, 44397, 44401-44407, 44409, 45355, 45378-45393, 45398, G6019, G6020, G6021, G6024, G6025, G0105, and G0121 |
|  | Excluded patients with history of IBD or a diagnosis of IBD at the date of initial CPT colonoscopy code | IBD ICD9 Codes | 555.0-555.2, 555.9, 556.0-556.6, 556.8, and 556.9 |
| Abnormal colonoscopy controls  (n=1,013,630) | Included those with any abnormal procedure codes 0 to 30 days post baseline | CPT codes for abnormal colonoscopy | 44388-44394, 44397, 44401-44407, 44409, 45355, 45379-45393, 45398, G6019, G6020, G6024, G6025, and G0105. |
| Normal colonoscopy cases  (n=825,413) | Included those with any diagnostic procedure codes at baseline  Excluded those with abnormal codes 0 to 30 days post baseline or a pathology report 0 to 30 days post baseline* | CPT codes for normal colonoscopy | 45378 and G0121 |
| *We clarify that for a subject to qualify as normal, they cannot have a document with abnormal colonoscopy within 30 days of their CPT colonoscopy date. Therefore, these subjects are shifted into the abnormal colonoscopy group if a subject has both CPT codes for normal and abnormal colonoscopy.  **Abbreviations:** CPT, Current Procedural Terminology; IBD, Inflammatory bowel disease; ICD9, International Classification of Diseases, 9^th^ Revision.. | | | |
